# Fructose:Glucose Ratios—A Study of Sugar Self-Administration and Associated Neural and Physiological Responses in the Rat

**DOI:** 10.3390/nu7053869

**Published:** 2015-05-22

**Authors:** AnneMarie Levy, Paul Marshall, Yan Zhou, Mary Jeanne Kreek, Katrina Kent, Stephen Daniels, Ari Shore, Tiana Downs, Maria Fernanda Fernandes, David M. Mutch, Francesco Leri

**Affiliations:** 1 Department of Psychology, University of Guelph, Guelph, ON N1G 2W1, Canada; E-Mails: alevy01@uoguelph.ca (A.L.); pmarshal@uoguelph.ca (P.M.); katekent15@gmail.com (K.K.); daniels@uoguelph.ca (S.D.); shorea@mail.uoguelph.ca (A.S.); tianadowns@hotmail.com (T.D.); mariafer@uoguelph.ca (M.F.F.); 2 Laboratory of Addictive Diseases, Rockefeller University, New York, NY 10065, USA; E-Mails: zhouya@mail.rockefeller.edu (Y.Z.); Mary.Jeanne.Kreek@rockefeller.edu (M.J.K.); 3 Department of Human Health & Nutritional Science, University of Guelph, Guelph, ON N1G 2W1, Canada; E-Mail: dmutch@uoguelph.ca

**Keywords:** fructose, glucose, self-administration, hypothalamus, nucleus accumbens, dopamine 2 receptor, mu opioid receptor, fatty acid, hepatic, rat

## Abstract

This study explored whether different ratios of fructose (F) and glucose (G) in sugar can engender significant differences in self-administration and associated neurobiological and physiological responses in male Sprague-Dawley rats. In Experiment 1, animals self-administered pellets containing 55% F + 45% G or 30% F + 70% G, and Fos immunoreactivity was assessed in hypothalamic regions regulating food intake and reward. In Experiment 2, rats self-administered solutions of 55% F + 42% G (high fructose corn syrup (HFCS)), 50% F + 50% G (sucrose) or saccharin, and mRNA of the dopamine 2 (D2R) and mu-opioid (MOR) receptor genes were assessed in striatal regions involved in addictive behaviors. Finally, in Experiment 3, rats self-administered HFCS and sucrose in their home cages, and hepatic fatty acids were quantified. It was found that higher fructose ratios engendered lower self-administration, lower Fos expression in the lateral hypothalamus/arcuate nucleus, reduced D2R and increased MOR mRNA in the dorsal striatum and nucleus accumbens core, respectively, as well as elevated omega-6 polyunsaturated fatty acids in the liver. These data indicate that a higher ratio of fructose may enhance the reinforcing effects of sugar and possibly lead to neurobiological and physiological alterations associated with addictive and metabolic disorders.

## 1. Introduction

The World Health Organization estimated that between 1980 and 2008, the rate of obesity (BMI > 30 kg/m^2^) doubled worldwide [1]. In Canada alone, the prevalence of obesity in adults has tripled between 1985 and 2011 [2]. These trends are a cause for serious concern, because obesity is causally linked to cardiovascular disease, type 2 diabetes, stroke, liver disease and cancer [3,4,5]. 

There are several factors that promote the development and maintenance of obesity, including interactions between genetic [6,7], metabolic [8], neurobiological [9], psychosocial [10,11] and economic [12] factors. Nevertheless, it is clear that one important component is nutrition, with particular reference to excessive consumption of foods containing high levels of fats [13] and sugars [14,15,16].

Within this context, there is the interesting issue of whether different sugars play a differential role in contributing to overeating and obesity. More specifically, it is proposed that fructose may be more “hazardous” than glucose because of its limited effects on cessation of food intake and on activation of brain regions that regulate this homeostatic response [17,18,19]. These observations have led to the hypothesis that sweeteners, such as high fructose corn syrup (HFCS), which contain a higher fructose to glucose ratio than sucrose, may be “addictive” and, thus, critically involved in hedonic overeating leading to obesity [20,21,22,23]. 

This hypothesis, however, has been debated, because it is unclear whether the behavioral and neural effects of pure fructose and pure glucose can accurately predict the effects of combinations of these same monosaccharaides at different ratios [24,25]. More specifically, is it possible that a 55% fructose-42% glucose ratio (typical of HFCS-55) can engender bio-behavioral effects that are significantly different from those of a 50%-50% ratio (typical of sucrose)? On the one hand, it has been reported that HFCS produces a smaller insulin response than sucrose in younger adults [26] and that it has more pronounced effects on weight gain and abdominal adiposity in rats compared to sucrose [14]. On the other hand, HFCS and sucrose consumption do not appear to elicit differences in short-term measures of satiety [27,28] or metabolic responses (*i.e.*, plasma levels of leptin and ghrelin [29])*.* Therefore, three complementary experiments were designed to address the question of whether differences in the ratio of fructose (F):glucose (G) in sugar impact reward-related behaviors, as well as associated neural and physiological responses.

## 2. Methods

### 2.1. Study Overview

In Experiment 1, non-food-restricted rats self-administered pellets containing 55% F + 45% G or 30% F + 70% G. The 55% F + 45% G pellet was selected to approximate the ratio found in HFCS-55, while the 30% F + 70% G pellet is the typical “sucrose” pellet that is employed in operant self-administration experiments. Immediately after the last self-administration session, brains were harvested and processed by immunohistochemistry to quantify the expression of Fos, a nuclear protein that indexes recent neuronal activation [30,31]. Fos was analyzed in three hypothalamic nuclei. The perifornical area of the lateral hypothalamus was selected because of its role in regulating food intake and its involvement in reward via dopaminergic and GABAergic projections to the nucleus accumbens [32,33]. The arcuate nucleus of the hypothalamus was selected because of its regulation of appetite by nutrient sensing hormones [34]. Finally, the dorsomedial hypothalamic nucleus was selected because of its role in mediating food consumption, as well as body weight regulation [35]. To measure possible differences in palatability, taste reactivity tests [36] were employed to compare hedonic orofacial responses to the two pellets.

In Experiment 2, food-restricted rats self-administered intraoral infusions of isocaloric solutions of HFCS-55 (55% F + 43% G) or sucrose (50% F + 50% G). Food-restriction was introduced to better control the quantity of food consumed prior to behavioral testing. Operant intraoral self-administration [37] was adopted to ensure that every operant response was followed by a sugar reward and to test two different schedules of reinforcement: continuous and progressive ratios. Following self-administration, a solution hybridization RNase protection-trichloroacetic acid (TCA) precipitation assay [38] was employed to quantify mRNA of dopamine D2 and mu-opioid (MOR) receptors in the striatum. These genes were selected because of their involvement in addictive behaviors and because their expression is altered by exposure to drugs of abuse [39,40]. The control group corresponded to rats that self-administered the non-caloric sweetener saccharin [41].

Finally, in Experiment 3, non-food restricted rats had *ad libitum* access to the same isocaloric solutions of HFCS-55 (55% F + 43% G) and sucrose (50% F + 50% G) in their home cages. Following the self-administration period, total adipose (epididymal; inguinal) and liver tissues were weighed. These adipose depots increase in mass in response to diet-induced obesity in rats [42,43]. Gas chromatography was also employed to quantify hepatic fatty acids. Polyunsaturated fatty acids omega-3 and omega-6 (PUFA; *n*-3 and *n*-6) were studied because of their involvement in regulating inflammatory processes associated with metabolic disorders and obesity [44].

### 2.2. Animals

Adult male Sprague-Dawley rats (Charles River, St-Constant, QC, Canada) weighing 225–250 g were used for all experiments. Rats were single-housed, maintained on a reverse light/dark cycle (7:00 a.m. lights off, 7:00 p.m. lights on) and given *ad libitum* access to rat chow and water in the home cage. Where indicated in the subsequent procedures, rats were food restricted for 18 h prior to each self-administration session and then fed chow *ad libitum* for 3 h immediately following the session. Behavioral testing occurred during the active (dark) phase. All experiments were approved by the Animal Care Committee of the University of Guelph and were carried out in accordance with the recommendations of the Canadian Council on Animal Care. 

### 2.3. Intraoral Surgery

Rats were surgically implanted with intraoral (IO) cannulas and head posts under general anesthesia with 5% isoflurane for induction and maintenance (for detailed descriptions of surgical procedures, please see [37]). Following surgery, IO cannulas were flushed once per day for three days with a solution of chlorhexidine (Ayerset, Fort Dodge, IA, USA). 

### 2.4. Apparatus

#### 2.4.1. Operant Self-Administration of Pellets

Self-administration of pellets was conducted in an automated apparatus (Med Associates, St. Albans, VT, USA) comprised of 8 arms radiating from a central octagonal hub. The central hub was equipped with eight automatically operated guillotine doors. A pellet receptacle was located at the end of each arm, and a photo beam situated inside detected nose pokes. A pellet dispenser was positioned behind each receptacle, located outside the arm (please see [45], for a detailed description of the apparatus and[46], for a schematic diagram). 

#### 2.4.2. Operant Self-Administration of Solutions

Twenty-six Plexiglas operant conditioning chambers (Model ENV-008CT, Med Associates, Lafayette, IN, USA), each enclosed in a larger sound-attenuating plywood cabinet (model ENV-018M, Med Associates), were employed. Each operant chamber had a house light (28 W), a cue light (28 W) and three levers. The “active” lever was retractable, was positioned 8 cm above the floor and 10 cm from the “inactive” lever. A second “inactive” lever was positioned 8 cm above the floor, on the opposite side of the chamber. The active lever was connected to an infusion pump (Razel Scientific Instruments, Stamford, CT) positioned outside of the sound-insulating cabinet. The cue light was located 3 cm above this lever and served as a conditioned stimulus (CS) paired with the delivery of intraoral infusions. The inactive levers served to control for baseline, non-reinforced operant behavior: pressing on either had no consequences, but all presses were recorded. 

#### 2.4.3. Home Cage Self-Administration

To measure HFCS and sucrose drinking in home cages, 100 mL no drip water bottles (Thermo Scientific, Waltham, MA, USA) with metal spouts (Ancare, Bellemore, NY, USA) secured within rubber stoppers (Fisher Scientific, Ottawa, ON, Canada) were placed in the home cage of rats alongside standard water bottles. 

#### 2.4.4. Taste Reactivity

The taste reactivity apparatus included a clear Plexiglas chamber (22.5 cm × 26.0 cm × 20.0 cm) with an opaque lid placed over a transparent glass surface. A digital video camera (Sony, DCR-HC48) was pointed at a mirror placed at a 45° angle under the glass surface to visualize the ventral surface of the rat and record orofacial reactions [47]. 

### 2.5. Food and Sugars

For all experiments, rats were fed a standard laboratory chow (18% protein and 4% fat; Teklad Global Diets, Harlan Laboratories). In Experiment 1, the solid pellets were purchased from Bio-Serv (45 mg Dustless Precision Pellets, Frenchtown, NJ, USA). Pellets contained: dextrose, fructose, cellulose and magnesium stearate. The nutritional profile of the pellets was: 0% fat, 0% protein, 89.5% carbohydrate (609 g/kg monosaccharaides, 285 g/kg disaccharides), <10% moisture and 3.8% fiber. Pellets differed in percent of monosaccharaides used: high ratio fructose = 55% F-45% G; low ratio fructose = 30% F-70% G. At these ratios, the caloric value of the pellets was nearly identical: high ratio fructose = 3.66 kcal/gram (0.165 kcal/pellet); low ratio fructose = 3.58 kcal/gram (0.161 kcal/pellet). 

For Experiments 2 and 3, solutions were made by diluting HFCS (HFCS-55 formula; Natures Flavors, CA, USA), sucrose (RedPath, ON, Canada) or saccharin (sodium salt hydrate 99+ %, ACROS Organics, NJ, USA) in reverse osmosis water. These solutions were stored at room temperature for the duration of the experiments. The nutritional profiles were: HFCS (serving of 4 g) = 0% fat, 0% protein and 0.26% carbohydrate; sucrose (serving of 4 g) = 0% fat, 0% protein and 0.1% carbohydrate. In Experiments 2 and 3, a solution of 25% HFCS was selected, because this concentration has been found to maintain robust operant intraoral self-administration [37]. The 20% sucrose solution was selected to match the caloric value of the 25% HFCS. More specifically, both 25% HFCS and 20% sucrose contained 0.75 kcal/mL. The non-caloric sweetener saccharin [41] was initially tested at 0.1%, because at this concentration, it elicits hedonic orofacial reactions in taste reactivity [48] and because rats spontaneously drink it from a bottle [49].

### 2.6. Behavioral Testing

#### 2.6.1. Operant Self-Administration of Pellets

In the three days prior to beginning self-administration in Experiment 1, 36 rats (not food restricted) were handled for 5 min/day and were randomly assigned to 55% F + 45% G or 30% F + 70% G pellets. During these three days, the groups (*n =* 18 each) received 20 pellets/day in their home cages. Each self-administration session began by placing the rat in the central hub of the apparatus for a 1-min adaptation period. Following adaptation, all guillotine doors were raised, and animals were tested for 10 min. During this period, rats obtained a pellet by entering any of the 8 arms and nose poking at the receptacle. Following this nose poke, the pellet dispenser became inactive; the animal was required to exit that arm, enter the central hub and then enter either the same or a different arm to obtain another pellet. In other words, animals were required to move from arm, to central hub, to arm in order to obtain successive rewards. At the end of each 10 min test, the receptacles were inspected, and the number of unconsumed sugar pellets was recorded. Testing was conducted once/day for 14 consecutive days, and rats were not food restricted for the duration of this experiment. Daily weight gain and chow consumption was measured and recorded approximately 2 h following each test session. Ninety minutes after the conclusion of the last self-administration session, brains were collected by euthanizing animals with an overdose of pentobarbital (Somnotol: 54.7 mg/kg, McGill University, Montreal, QC, Canada) and by intracardiac infusion with 0.9% saline and 4% paraformaldehyde. In order to interpret the Fos immunoreactivity data, a control group was also included (*n =* 11). These animals were transported and kept in the testing room according to the time line described above, but were not exposed to the maze or to the sugar pellets. Their brains were collected 90 min following the 14th day of exposure to the testing room. Finally, a separate group of rats (*n =* 8; not food restricted) were tested for taste reactivity to measure hedonic orofacial reactions in response to infusions of solutions sweetened with the pellets tested above. 

#### 2.6.2. Operant Intraoral Self-Administration

In the five days prior to beginning self-administration in Experiment 2, 32 rats (food restricted) were handled for 5 min/day and were randomly assigned to either 25% HFCS (55% F + 45% G) or 20% sucrose (30% F + 70% G) solutions. Prior to the beginning of self-administration, rats received a single taste reactivity test to measure hedonic orofacial reactions in response to the sugar solutions. Isocaloric solutions of HFCS or sucrose were self-administered daily during sessions lasting 3 h each. Sessions began with the activation of a house light, entry of the active lever and illumination of the conditioned stimulus (CS) light above it for 30 s. Subsequently, presses on the active lever resulted in a 2.5-s intraoral infusion of 80 µL of HFCS or sucrose (caloric content = 0.06 kcal/infusion). A time-out period of 27.5 s was imposed to allow sufficient time for ingestion of the intraoral infusion. During this period, the active lever was retracted, and the CS light was activated. No limit was imposed on the number of infusions obtainable within each session. 

Animals were trained to lever press on both continuous (fixed ratio 1 (FR1)) and progressive ratio (PR) schedules of reinforcement. On the PR schedule, the number of responses required to obtain each successive infusion increased exponentially within the session according to the formula: (5 ^e(injection number × 0.2)^) [50]. The breakpoint was indexed by the last infusion received prior to cessation of responding on the active lever for at least 1 h [50]. Rats self-administered 25% HFCS or 20% sucrose for a total of 47 sessions, and two PR tests were performed on Days 11 and 21 of testing. On Day 48, animals were euthanized, and brains were collected for mRNA quantification. In order to interpret the results of the mRNA analyses, an additional group of rats (*n =* 26) was trained to lever press for intraoral infusions of 0.1% saccharin, a non-caloric, but sweet solution, on an FR1 schedule of reinforcement under identical conditions. These animals were tested for 17 days, but because self-administration was minimal, additional concentrations were tested over 3 additional days. The number of infusions obtained did not increase; therefore, behavioral testing was terminated after 20 days of testing, and brains were collected on Day 21. 

#### 2.6.3. Home Cage Self-Administration

In the three days prior to beginning home cage self-administration in Experiment 3, 16 rats (not food restricted) were handled for 5 min/day and were randomly assigned to drink either 25% HFCS (55% F + 45% G) or 20% sucrose (30% F + 70% G). These animals were provided with *ad libitum* access to rat chow, water and either HFCS or sucrose solutions in their home cage for 45 consecutive days. At the same time every day, body weight, chow, water and sugar consumption were measured and recorded. On Day 46, animals were euthanized, adipose and liver tissues weighed and hepatic fatty acid composition measured by gas chromatography.

#### 2.6.4. Taste Reactivity

Taste reactivity (TR) testing [36,47,48] occurred over 2 days prior to beginning self-administration in Experiments 1 and 2. In Experiment 1, the pellets were dissolved in water (0.5 mL/pellet) using a magnetic stir bar and hot plate. Rats were individually placed in the test chambers for a 3-min habituation period. During this period, the intraoral cannula was attached to an infusion pump, and water was infused at a rate of 0.5 mL/min. Twenty-four hours later, rats were returned to the chamber, attached to the infusion pump and infused with the test solution at a rate of 0.5 mL/min. Orofacial reactions were recorded for the duration of the 3-min test, and behavior was subsequently scored using The Observer (Noldus Information Technology, Sterling, VA, USA). The frequency of forward (extensions of the tongue out the front of the mouth) and lateral (extensions of the tongue out the sides of the mouth, sweeping along the lips) tongue protrusions were scored and employed as the index of palatability [48]. In Experiment 2, identical procedures were employed to test the different sugar solutions self-administered (*i.e.*, 25% HFCS, 20% sucrose and 0.1% saccharin). 

### 2.7. Fos Immunoreactivity

In Experiment 1, brains were processed for Fos immunoreactivity (for detailed descriptions of the procedures, please see [51]. For each image captured (Leica software V3.6, Leica Microsystems Inc., Concord, ON, Canada), Fos immunoreactive cells were counted, divided by the area, and a mean density value ((count/um^2^) × 10^6^) per animal was calculated from 4–6 images from multiple bilateral slices using ImageJ (National Institutes of Health, Bethesda, MD, USA). Fos density was quantified by researchers blind to treatment groups.

The Paxinos and Watson (2004) brain atlas was used to identify locations of interest in reference to bregma, as well as changes in shape or size of areas analyzed for Fos immunoreactivity. Specifically, to determine where slices were in reference to bregma (ranging between −2.52–−3.12) for the hypothalamus, the size and shape of the hippocampus and lateral ventral were used, as well as the size and location of the optic tract [52]. Once bregma was determined, the perifornical areas of the lateral hypothalamus were localized with reference to the fornix, the dorsomedial hypothalamus was located around the crest of the third ventricle and the arcuate nucleus was drawn around its base. 

### 2.8. Solution Hybridization RNase Protection-TCA Precipitation

Following collection, brains were stored at −80 °C. The caudate-putamen and core/shell subdivisions of the nucleus accumbens were dissected on ice for subsequent mRNA quantification. For detailed descriptions of the solution hybridization RNase protection-TCA precipitation protocol, please see [38]. The mRNA levels for D2R and MOR were quantified (in attomoles/µg) in specific regions on the basis of known levels of constitutive expression, as well as previous results showing expression changes as a result of exposure to cocaine [40]. 

### 2.9. Gas Chromatography

Following collection, livers were stored at −80 °C. The frozen tissue (0.05 g of tissue from each lobe of the liver) was thawed on ice prior to fatty acid extraction (for detailed descriptions of the procedures, please see [53]. Fatty acid peaks were identified by comparison to retention times of fatty acid methyl ester standards, and fatty acid levels were determined quantitatively from the internal standard and expressed as μg/liver.

### 2.10. Statistical Analyses

Data reported in this manuscript were analyzed by mixed analysis of variance (ANOVA) and *t*-tests. Significant interactions and main effects were analyzed by multiple comparisons using the Student–Newman–Keuls method (α = 0.05). The exact values of non-significant results were not reported, and all analyses were performed using SigmaStat (v. 3.5 for Windows, SPSS Inc., NY, USA).

## 3. Results

### 3.1. Experiment 1

#### 3.1.1. Taste Reactivity

The mean frequency of tongue protrusions in response to infusions of solutions made by dissolving 55% F + 45% G or 30% F + 70% G pellets did not differ significantly (mean ± SEM = 72.6 ± 10.1 and 70.8 ± 14.1, respectively). 

#### 3.1.2. Operant Self-Administration of Pellets

**Figure 1:**
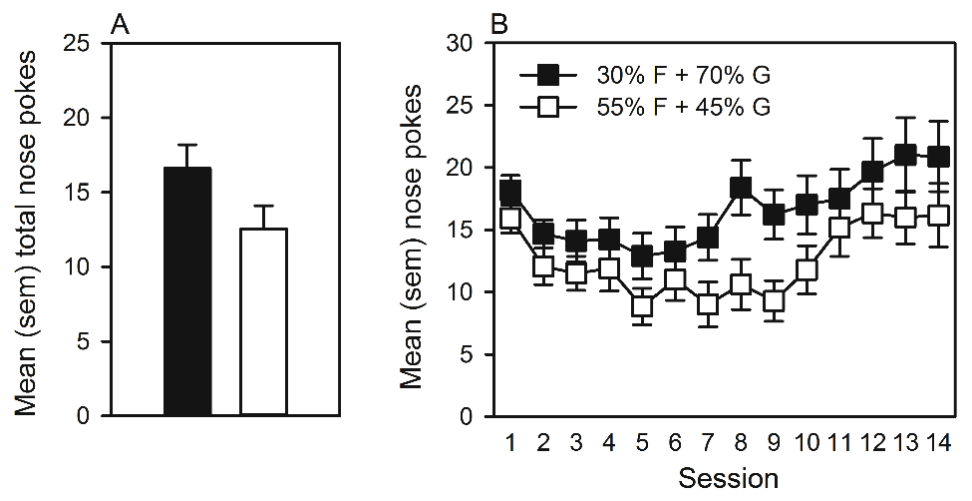
(**A**) Mean (SEM) total number of nose pokes emitted for 55% F (fructose) + 45% G (glucose) or 30% F + 70% G pellets. (**B**) Mean (SEM) daily number of nose pokes emitted over 14 sessions of self-administration for 55% F + 45% G or 30% F + 70% G pellets.

The mean total number of nose pokes emitted during 14 self-administration sessions was not statistically different between groups (Figure 1A). Although rats that self-administered 55% F + 45% G pellets nose poked less over the testing period (Figure 1B), the ANOVA revealed only a significant main effect of session (*F*(13, 442) = 10.97, *p <* 0.001). Moreover, during the 14 days of testing, no significant differences in daily consumption of chow or weight gain were observed between groups (data not shown). 

**Figure 2:**
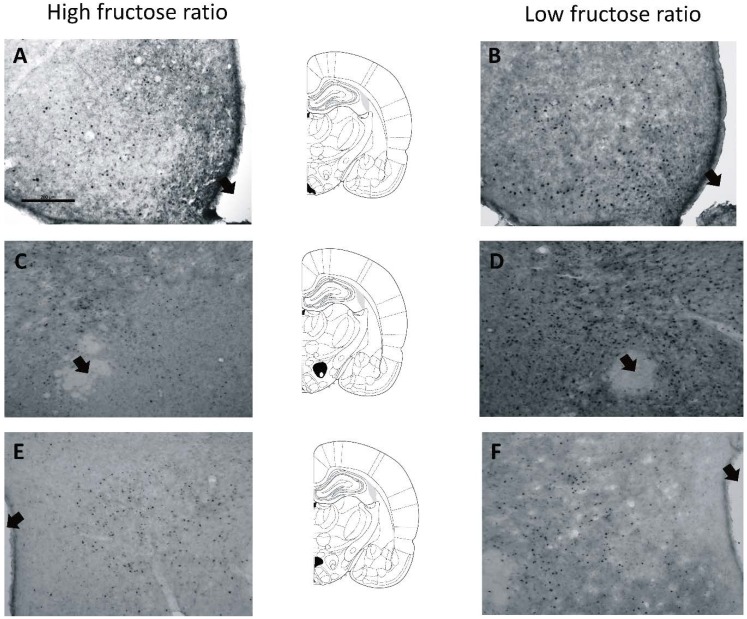
Representative microphotographs of c-Fos (calibration line = 200 μm) in the arcuate nucleus hypothalamus of rats that self-administered 55% F + 45% G (**A**) or 30% F + 70% G (**B**), perifornical area of the lateral hypothalamus of rats that self-administered 55% F + 45% G (**C**) or 30% F + 70% G (**D**), and dorsomedial hypothalamusof rats that self-administered 55% F + 45% G (**E**) or 30% F + 70% G (**F**). The black arrows indicate the location of the third ventricle (A,B,E,F) and fornix (C,D). Between each panel, diagrams from Paxinos and Watson (2004) with selected structures are blackened.

**Figure 3:**
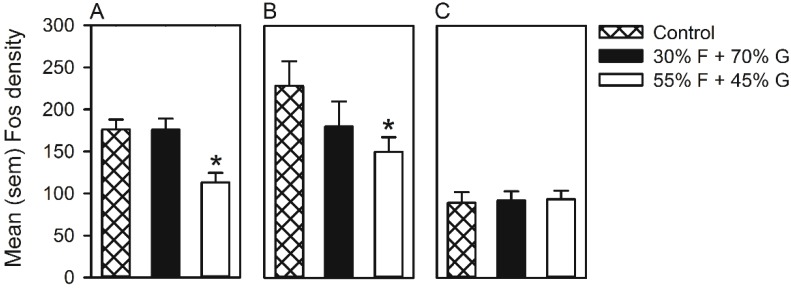
Mean (SEM) Fos density measured in the perifornical area of the hypothalamus (**A**), arcuate nucleus (**B**) and dorsomedial hypothalamus (**C**) of rats that self-administered 55% F + 45% G or 30% F + 70% G pellets. * Significant difference from the control group.

Representative microphotographs of hypothalamic regions selected for immunohistochemistry are included in Figure 2A–F. Mean Fos density in control, 55% F + 45% G and 30% F + 70% G groups is represented in Figure 3A–C. In the perifornical area of the lateral hypothalamus (Figure 3A), the ANOVA was significant (*F*(2, 33) = 8.08, *p <* 0.001), and multiple comparisons indicated that Fos density was significantly lower in rats that self-administered 55% F + 45% G. Within the arcuate nucleus (Figure 3B), Fos density was not significantly different between groups. However, *a priori* planned comparisons revealed that, compared to the control group, Fos density was significantly lower in rats that self-administered 55% F + 45% G (*t*(19) = 2.2, *p <* 0.05). Finally, in the dorsomedial hypothalamus (Figure 3C), no significant differences in Fos density were observed. 

### 3.2. Experiment 2

#### 3.2.1. Taste Reactivity

The mean frequency of tongue protrusions measured in response to infusions of 25% HFCS, 20% sucrose or 0.1% saccharin did not differ significantly (mean ± SEM = 130.6 ± 10.1, 121.8 ± 14.1, 118.8 ± 14.1, respectively). 

#### 3.2.2. Operant Intraoral Self-Administration

**Figure 4:**
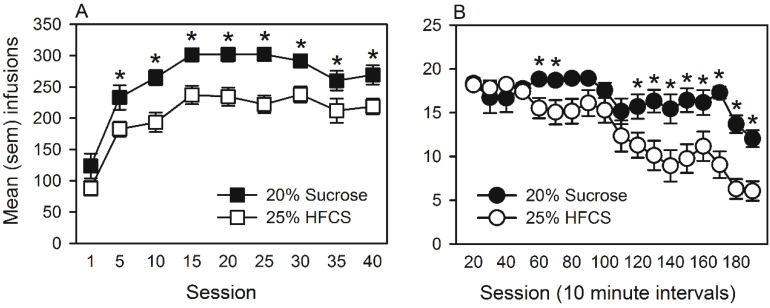
(**A**) Mean (SEM) infusions obtained on a fixed ratio one schedule of reinforcement by rats that self-administered isocaloric infusions of 25% high fructose corn syrup (HFCS) or 20% sucrose. * Significant difference between groups. (**B**) Mean (SEM) infusions of 25% HFCS and 20% sucrose obtained over time (ten-minute intervals) within self-administration Session 20. * Significant difference between groups.

The mean number of infusions obtained by lever pressing on an FR1 schedule for 25% HFCS or 20% sucrose across the self-administration period is represented in Figure 4A. The ANOVA revealed significant main effects of group (*F*(1, 240) = 16.78, *p <* 0.001) and of session (*F*(8, 240) = 46.5, *p <* 0.001). Multiple comparisons indicated that self-administration of HFCS was significantly lower than sucrose for Sessions 5–40. To understand this difference, the patterns of lever pressing for HFCS and sucrose were compared within a self-administration session in the middle of the experiment (*i.e.*, Session 20; Figure 4B). The ANOVA revealed a significant group by session interaction (*F*(17, 510) = 3.8, *p <* 0.001), as well as significant main effects of group (*F*(1, 510) = 12.9, *p <* 0.001) and of session (*F*(17, 510) = 14.3, *p <* 0.001). Multiple comparisons indicated that HFCS and sucrose self-administration differed primarily in the latter half of each session. When these same animals were tested on the PR schedule, the ANOVA revealed no significant group differences in breakpoints achieved on the first (mean ± SEM; HFCS = 10.9 ± 0.4; sucrose = 10.3 ± 0.6) or the second (HFCS = 9.0 ± 0.5; sucrose = 10.4 ± 0.6) PR tests. Finally, over the duration of the self-administration period, no significant group differences emerged in the daily consumption of chow, weight gain or total caloric intake (chow and sugar combined; data not shown). 

In a separate group of rats, self-administration of 0.1% saccharin was minimal (Table 1), and no significant differences in number of infusions across sessions were observed. Likewise, no significant differences in patterns of self-administration over time (10 minute intervals) were noted (data not shown). Finally, self-administration did not change when the concentration of saccharin was varied (Table 1). 

**Table 1:** Mean (SEM) infusions obtained on a fixed ratio one schedule of reinforcement by rats that self-administered infusions of 0.1% (self-administration (SA) Sessions 1–15), 0.01%, 1% and 10% saccharin.

	Concentration (%) of saccharin						
	0.1%						
SA session	1	5	10	15	0.01%	1%	10%
Infusions	27.7 (5.5)	47.4 (11.7)	39.5 (8.7)	31.2 (7.5)	18.9 (7.5)	16.0 (4.4)	15.8 (2.2)

**Figure 5:**
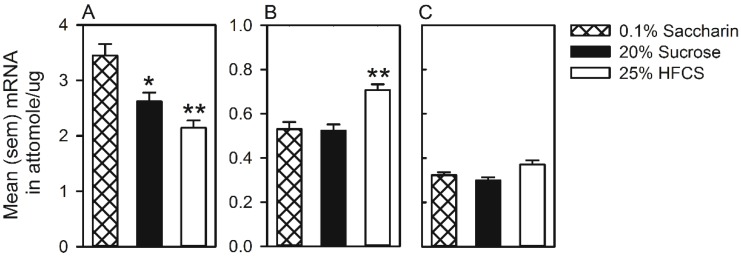
Mean (SEM) mRNA (attomole/μg) measured in the dorsal striatum (**A**), nucleus accumbens core (**B**) and nucleus accumbens shell (**C**) of rats that self-administered 25% HFCS, 20% sucrose or 0.1% saccharin. * Significant difference from 0.1% saccharin groups; ** significant difference from the 0.1% saccharin and 20% sucrose groups.

Figure 5 represents D2R mRNA expression in the dorsal striatum (Figure 5A) and MOR mRNA expression in the nucleus accumbens core (Figure 5B) and shell (Figure 5C) following self-administration of 25% HFCS, 20% sucrose and 0.1% saccharin. In the dorsal striatum, the ANOVA on D2R mRNA expression was significant (*F*(2,22) = 13.3, *p <* 0.001), and multiple comparisons indicated that expression was significantly lower in animals that self-administered HFCS, in comparison to both sucrose and saccharin. In the nucleus accumbens core, the ANOVA was also significant (*F*(2,20) = 13.0, *p <* 0.001), and multiple comparisons indicated that MOR mRNA was significantly higher in rats that self-administered HFCS, in comparison to both sucrose and saccharin. The ANOVA on MOR mRNA expression in the nucleus accumbens shell was significant (*F*(2,20) = 5.5, *p <* 0.01), and multiple comparisons revealed that expression differed between the HFCS and the sucrose groups only. Finally, MOR mRNA expression was also quantified in the dorsal striatum, but no significant differences were found (mean ± SEM mRNA in attomole/µg: HFCS = 0.17 ± 0.01; sucrose = 0.17 ± 0.01; saccharin *=* 0.16 ± 0.01). 

### 3.3. Experiment 3

#### Home Cage Self-Administration

**Figure 6:**
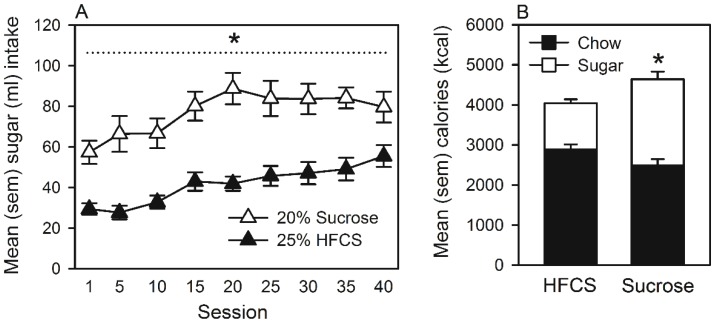
(**A**) Mean (SEM) sugar (mL) intake by rats that self-administered 25% HFCS or 20% sucrose in their home cages. * Significant difference between groups. (**B**) Mean (SEM) total calories (kcal) consumed by rats. The proportions of calories derived from chow and sugar consumption are represented by the black and white stacked bars, respectively. * Significant difference in total caloric intake between the 25% HFCS and 20% sucrose groups.

The mean volume of 25% HFCS or 20% sucrose self-administered by drinking from a bottle in the home cage is represented in Figure 6A. The ANOVA revealed significant main effects of group (*F*(1, 8) = 24.6, *p <* 0.001) and of session (*F*(8, 112) = 13.7, *p <* 0.001). Similar to Experiments 1 and 2, self-administration of HFCS was significantly lower than that of sucrose. Figure 6B represents total caloric intake over the entire self-administration period, which was significantly lower in the HFCS group (*t*(14) = 3.1, *p <* 0.01). Additionally, Figure 6B represents the proportion of total caloric intake accounted for by chow or by sugar consumption, and the ANOVA revealed a significant group by food interaction (*F*(1, 14) = 13.8, *p <* 0.01), as well as significant main effects of group (*F*(8, 14) = 9.5, *p <* 0.01) and of food (*F*(1, 14) = 29.9, *p <* 0.001). Multiple comparisons indicated that the overall group difference in total caloric intake was the result of differences in sugar, but not chow consumption. That is, the proportion of calories derived from sugar was significantly lower in rats drinking HFCS, while no significant group differences in calories derived from the consumption of chow were observed. 

**Figure 7:**
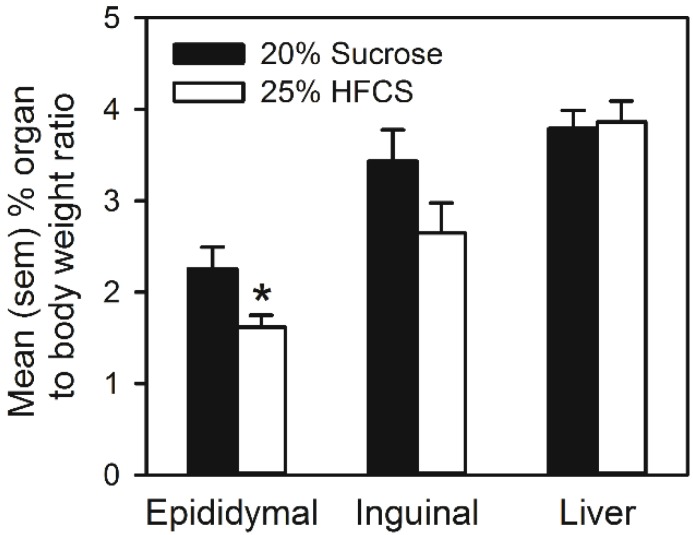
Mean (SEM) percent organ to body weight ratio of epididymal fat pads, inguinal fat pads, as well as whole livers in rats that self-administered 25% HFCS or 20% sucrose in home cages. * Significant difference between groups.

Figure 7 represents the mean % organ to body weight ratio of epididymal fat pads, inguinal fat pads, as well as whole livers following sugar self-administration. Epididymal fat pads were significantly smaller in rats that self-administered HFCS (*t*(14) = 2.3, *p <* 0.05), while no significant group differences were noted in inguinal fat pads or livers. 

**Figure 8:**
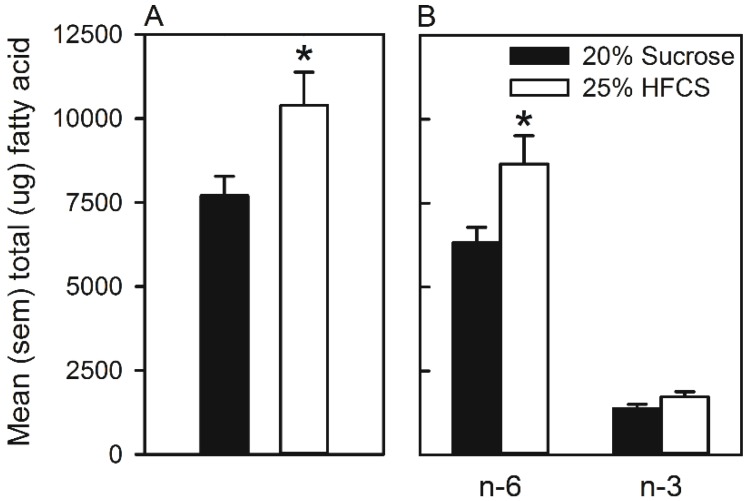
(**A**) Mean (SEM) total fatty acids (µg) measured in the livers of rats that self-administered 25% HFCS or 20% sucrose in home cages. (**B**) Mean (SEM) polyunsaturated fatty acids *n*-6 and *n*-3 measured in the livers of rats that self-administered 25% HFCS or 20% sucrose in home cages. * Significant difference between groups.

Figure 8A represents the mean total fatty acids measured in livers. Overall, the fatty acid concentration was significantly higher in the livers of rats that self-administered HFCS (*t*(14) = 2.4, *p <* 0.05). More specifically, when the PUFA *n*-6 and *n*-3 were measured (Figure 8B), the ANOVA revealed a significant group by PUFA interaction (*F*(1, 14) = 6.5, *p <* 0.05), as well as significant main effects of group (*F*(1, 14) = 5.6, *p <* 0.05) and of PUFA (*F*(1, 14) = 228.4, *p <* 0.001). Multiple comparisons indicated that the level of hepatic *n*-6 was significantly higher in rats that self-administered HFCS, while no group difference in the level of *n*-3 was observed.

## 4. Discussion

Three experiments were performed in rats to investigate whether sugars composed of different fructose (F):glucose (G) ratios engender differences in self-administration, as well as associated neurobiological and physiological responses. Across all experiments, it was found that a higher ratio of fructose was associated with reduced sugar self-administration. This behavioral difference could not be attributed to factors inherent to the procedures employed (*i.e.*, length of self-administration sessions, mode of delivery or quantity of sugar consumed), nor to the nutritional status of the animals at the time of testing (*i.e.*, food restricted or not food restricted). Interestingly, exposure to the high fructose ratio sugar was also associated with lower Fos expression in the perifornical area of the lateral hypothalamus and arcuate nucleus, reduced expression of D2R mRNA in the dorsal striatum and elevated MOR mRNA in the core of the nucleus accumbens. Similar neural alterations have been observed following exposure of drugs of abuse [39,40,54]. Finally, in these same animals, hepatic *n*-6 PUFA were elevated, and this has been associated with chronic inflammatory states characteristic of metabolic diseases and obesity [44,55]. 

In Experiment 1, non-food-restricted rats self-administered pellets containing 30% F + 70% G or 55% F + 45% G, and 90 min after the last self-administration session, their brains were processed for Fos immunoreactivity in hypothalamic regions involved in the regulation of food intake and reward. In self-administration, rats tended to emit fewer nose pokes for high ratio fructose pellets. Although not statistically different, this observation was in the same direction of the significant behavioral effects reported in Experiments 2 and 3, following HFCS and sucrose self-administration. In the analysis of Fos immunoreactivity, compared to the control group, the intake of the high ratio fructose pellets was associated with a significant reduction in Fos density in the perifornical area of the lateral hypothalamus and arcuate nucleus (see Figure 3A,B). These hypothalamic regions contain cells that synthesize and release peptides regulating food reward and food intake, including neuropeptide Y [56], melanocortins [57], cocaine- and amphetamine-regulated transcript [58] and orexins [59]. While the phenotype of the Fos-expressing cells was not identified [60], these data are in line with the observations that fructose is generally less effective than glucose in suppressing activity in the hypothalamus and other brain regions [18]. Interestingly, the taste reactivity test indicated that palatability or “liking” [61] of the pellets was not different, suggesting that the difference in Fos activity was not related to differences in the taste of the pellets. 

In Experiment 2, food-restricted rats self-administered isocaloric solutions of 25% HFCS or 20% sucrose on FR1 and PR ratio schedules of reinforcement, and brains were processed for mRNA expression of genes associated with addictive behaviors. Self-administration of HFCS was significantly lower than sucrose on the FR1 schedule (see Figure 4A). The pattern of self-administration behavior within Session 20 indicated that initial (60 min; Figure 4B) lever pressing was indistinguishable between groups. However, HFCS self-administration declined, while sucrose self-administration remained elevated in the last 2 h of each session (180 min in total). In contrast, the breakpoints achieved [50] on the PR schedule did not differ between groups or across PR tests (Sessions 11 and 21). It is unlikely that the observed group difference on the FR1 schedule was due to differences in satiety levels. In fact, food restriction controlled the quantity of food consumed in the hours prior to self-administration. Likewise, following each session, no significant differences in daily chow consumption or growth emerged. The results of the taste reactivity test also indicated that the group difference was not due to differences in palatability, as orofacial reactions did not differ in response to solutions made with either 25% HFCS or 20% sucrose. 

Compared to HFCS and sucrose, self-administration of the non-caloric 0.1% saccharin solution [41] did not support self-administration or engender differences in the pattern of self-administration within sessions. As a matter of fact, a wide range of saccharin concentrations (see Table 1) could not maintain self-administration at the same level of HFCS or sucrose, indicating that these sugars are reinforcing because of their caloric content. The saccharin findings were not particularly surprising in light of data suggesting that this sweetener has weak reinforcing properties in rats [62] and weak effects on dopamine release in the nucleus accumbens [63,64]. Furthermore, the minimal responding observed during saccharin self-administration could not be attributed to differences in taste reactivity, as orofacial reactions in response to infusions of saccharin were statistically indistinguishable from those made in response to HFCS and sucrose. 

Therefore, given that the sugar solutions were isocaloric, perceived as equally palatable and the nutritional status of animals at the time of testing was controlled, these data suggest that HFCS may be a more potent reinforcer than sucrose. This is consistent with the observation that increasing the concentration of reinforcing stimuli, such as drugs of abuse [65,66], reduces self-administration on FR1 schedules. Although breakpoint achieved on PR tests is also employed as an index of the reinforcing efficacy of stimuli [50], this schedule may not have been sensitive enough to detect differences in the reinforcing properties of the sugars employed in the current experiment given the limited number of reinforcers obtained. This is a likely interpretation, as group differences were only revealed in the latter half of the self-administration sessions on FR1 schedules. Moreover, caloric content appears to play a principal role in determining the breakpoint achieved. That is, our laboratory has previously reported that as the caloric content and concentration of HFCS increases, the breakpoint achieved in self-administration also increases [37]. Likewise, increasing the caloric content and concentration of sucrose engenders similar increases in break points [67]. Therefore, it is possible that in the current experiment, group differences in breakpoints achieved were not observed because isocaloric solutions of HFCS and sucrose were employed. 

The results of the mRNA analysis indicated that even though less HFCS was self-administered compared to sucrose, alterations in the expression of reward-related genes in two regions of the brain implicated in addictive behaviors were specific to HFCS intake. HFCS self-administration reduced the expression of D2R mRNA in the dorsal striatum compared to both sucrose and saccharin controls (see Figure 5A). Sucrose self-administration also reduced the expression of D2R mRNA in the dorsal striatum, but this was only different from rats that self-administered saccharin. In the dorsal striatum, expression of D2R mRNA, as well as D2R availability is reduced by repeated consumption of cafeteria diets [68], sucrose [69] and exposure to drugs of abuse [39,40,54]. These neural adaptations are associated with deficits in reward sensitivity, as well as enhanced impulsive [54] and compulsive [68] behaviors. Sucrose self-administration has been found to reduce the expression of D2R mRNA in the dorsal striatum, not only in the current experiment, but also other studies [69]. However, to our knowledge, this is the first report of similar effects following HFCS self-administration and of significant differences between HFCS and sucrose. 

Elevated MOR mRNA in the nucleus accumbens core was also observed following HFCS, but not sucrose or saccharin self-administration. This region is critically involved in the attribution of incentive motivation to stimuli during instrumental and Pavlovian learning [70,71,72], and similar neural adaptations were reported following repeated administration of drugs of abuse, such as cocaine [40,73]. However, it should be noted that the interpretation of these results is limited to protein biosynthesis and/or release, because the protein levels were not measured. 

In Experiment 3, bottles of either 25% HFCS or 20% sucrose were placed alongside water and chow in the home cages of rats, and following self-administration, adipose and liver tissue were weighed and the composition of fatty acids in the liver measured by gas chromatography. Once again, even if the solutions were isocaloric and animals were not food restricted, it was found that HFCS engendered lower self-administration than sucrose (see Figure 6A). Over the duration of the experiment, total caloric intake was lower in rats that self-administered HFCS (see Figure 6B). This difference was attributable to group differences in sugar intake. That is, the proportion of calories derived from chow consumption did not differ between HFCS and sucrose groups; however, the proportion of calories derived from sugar consumption was significantly lower in rats self-administering HFCS (see Figure 6B). This caloric discrepancy was associated with differences in the accumulation of adipose tissue: epididymal (but not inguinal) fat pads of rats self-administering sucrose were significantly larger (see Figure 7). Similar changes in epididymal fat pads have been reported in rats maintained on high caloric cafeteria diets [42]. 

Interestingly, self-administration of HFCS increased the level of hepatic *n*-6 fatty acids. The level of *n*-3 was not altered (Figure 8). *n*-3 (commonly derived from fish oils) is anti-inflammatory and protects against, as well as helps recover, loss of functions associated with insulin resistance, non-alcoholic liver and cardiovascular diseases, as well as low-grade inflammatory states characteristic of obesity [74,75,76,77]. In contrast, diets rich in *n*-6 (commonly derived from corn oils or soybeans; [74]) contribute to these pathologies, firstly, because they are utilized in the synthesis of pro-inflammatory molecules (for a review, please see [44,55]) and, secondly, by suppressing anti-inflammatory responses. Therefore, the results of Experiment 3, and other studies [78], lend support to the hypothesis [44] that frequent consumption of HFCS may be a risk factor for developing diseases that are characterized by chronic inflammatory states.

## 5. Conclusions

These self-administration experiments in rats provide converging evidence that differences in fructose:glucose ratios can have a significant impact on self-administration behaviors. It is likely that sugars containing a higher ratio of fructose engendered lower self-administration, because this combination has more potent reinforcing effects. The subsequent brain and liver tissue analysis supported this interpretation. Altered neural activity in regions of the hypothalamus responsible for food reward, gene expression changes putatively associated with addiction and accumulation of fatty acids in the liver associated with inflammatory responses were specific to HFCS self-administration and occurred despite reduced consumption in comparison to sucrose. In other words, it took less exposure to sugars with a higher ratio of fructose to glucose to engender both neural and physiological changes associated with addictive and metabolic diseases. Future intraoral self-administration studies employing isocaloric solutions of pure glucose and pure fructose will be necessary to elucidate the relative contribution of these monosaccharides to reinforcement (it is not possible to manufacture pure glucose or pure fructose sugar pellets). Furthermore, in addition to 55% fructose and 42% glucose, HFCS contains 3% glucose polymers (polycose; [79]). Because polycose contributes to the reinforcing efficacy of HFCS [79], it should likewise be tested on its own in future studies of sugar self-administration. 
